# The characteristic, antioxidative and multiple organ protective of acidic-extractable mycelium polysaccharides by *Pleurotus eryngii* var. *tuoliensis* on high-fat emulsion induced-hypertriglyceridemic mice

**DOI:** 10.1038/s41598-018-35706-8

**Published:** 2018-11-30

**Authors:** Zheng Gao, Qiangqiang Lai, Qihang Yang, Nuo Xu, Wenbo Liu, Fulan Zhao, Xinchao Liu, Chen Zhang, Jianjun Zhang, Le Jia

**Affiliations:** 1 College of Life Science, Shandong Agricultural University, Taian, 271018 PR China; 2 The First People’s Hospital of Taian, Taian, 271000 PR China

## Abstract

The antioxidant and multiple organ protection effects of acid- extracted mycelia polysaccharides (Ac-MPS) from *Pleurotus eryngii* var*. tuoliensis* on HFE-induced hypertriglyceridemic mice were investigated. The results showed that Ac-MPS have potential ability to relieve the hypertriglyceridemia and preventing oxidative stress by decreasing levels of TG, TC LDL-C, elevating contents of HDL-C in serum, increasing the activities of SOD, GSH-Px, CAT and T-AOC, and the down regulating MDA and LPO contents in liver, heart, kidney and spleen. And the histopathological observations also displayed that Ac-MPS could alleviate organ damage. Moreover, the GC, HPGPC, FT-IR and AFM analyses revealed the Ac-MPS possessed the typical polysaccharides structure with the molecular weights (Mw) of 2.712 × 10^5^ Da. These conclusions indicated that the Ac-MPS had the potential to develop new drugs for hypertriglyceridemia-induced multiple organ failure.

## Introduction

The hypertriglyceridemia (HTG), clinically reflected by fasting blood triglyceride (TG) levels over than 150 mg/dL, is a common lipid metabolic disorder disease with possibly pathogenic mechanisms including internal genetic factors as well as external factors of obesity, excess alcohol intake, presence of metabolic syndrome, insulin resistance, medications, pregnancy, endocrine diseases and autoimmune diseases^1^. And with the changes of diet structure, an increasing number of people have suffered hyperlipidaemia worldwide. Besides, according to previous literatures, many clinical complications containing cardiovascular diseases, diabetes, neuropathy, pancreatitis, and insufficiency on organs could be induced by severe HTG, subsequently^2,3,4^. Despite considerable improvements in medical care, the hypertriglyceridemic remains one of the most human health challenge^5^. Clinically, the main tool for treating the HTG was lifestyle interventions and pharmacotherapies such as statins, ezetimibe, and niacin thus far. However, these treatments are flawed partly owing to the unexpected side-effects^6^. The natural substance of polysaccharides from edible and medicinal mushrooms had been received more and more clinical attentions contributing to the potentially therapeutic effects on hypertriglyceridemic-related diseases^7^. Hence, it is quite necessary to explore safe and effective anti-HTG agents for providing an alternative option for preventing and treating HTG and its complications. Increasing evidences have demonstrated that the severe HTG was associated with a systemic oxidative stress response, produced by the over-production of reactive oxygen species (ROS) containing superoxide anion radicals (O_2_^−^•), hydroxyl radicals (•OH), and 1,1-dipheny-l-2-picrylhydrazyl (DPPH) radicals^8,9^. Meanwhile, the over-plus ROS paly vital roles in accelerating the organs damages induced by HTG^9^. Thus, it seems necessary on remitting the HTG-induced-organic-insufficiency by inhibiting the production of ROS.

Recently, mushrooms had drawn greater clinical attentions due to their excellent mouthfeel and healthy properties. And the *Pleurotus eryngii* var. *tuoliensis*, one of the most popular edible mushrooms, had been demonstrate on showing abundant bioactive components including proteins, polysaccharides, trace elements and fat^10^. The *P. eryngii* var. *tuoliensis* was applied in functional foods ordinarily^11^. Previous literatures have demonstrate that the polysaccharides extracted from *P. eryngii var. tuoliensis* usually showed various biological activities, such as antioxidant, immunomodulatory and protective effects on hepatocytes^12,13^. Furthermore, the submerged fermentation technique with the short period, high yield and high-purity, is widely applied in the production of mycelia^14^ and provide a useful applications for the production of rare mushroom. Moreover, the assistant methods of enzymatic, acidic and alkalic in traditional extraction process have been demonstrate beneficial for the production and quality of polysaccharides owing to the changes of physicochemical properties^15^. However, fewer literatures about acidic-assisted method has been applied in the extraction of *P. eryngii* var. *tuoliensis* polysaccharides. Thus, it is a desirable need for exploring the acidic-extractable mycelia polysaccharides (Ac-MPS) from *P. eryngii* var. *tuoliensis*.

This work was designed to investigate the anti-hypertriglyceridemic, antioxidant and organic protection of Ac-MPS on hypertriglyceridemic mice induced by high-fat emulsion (HFE). In addition, the preliminary structure of Ac-MPS was also assayed.

## Materials and Methods

### Strain and chemicals

The strain of *P. eryngii* var. *tuoliensis* used in this experiment was provided by Fungi Institute of Academy of Agricultural Sciences (Taian, China). The diagnostic kits for analyzing the activities of superoxide dismutase (SOD), glutathione peroxidase (GSH-Px), catalase (CAT), and total antioxidant capacity (T-AOC), and the contents of lipid peroxide (LPO) and malondialdehyde (MDA) were from Nanjing Jiancheng Bioengineering Institute (Nanjing, China). Eight standard monosaccharide include arabinose (Ara), fucose (Fuc), galactose (Gal), glucose (Glu), xylose (Xyl), rhamnose (Rha), ribose (Rib), and mannose (Man) were from Sigma Chemicals Company (St. Louis, USA). DEAE-52 cellulose was provide by Pha. Co. (New Jersey, USA). All other chemicals used in present work were analytical grade and purchased from local chemical suppliers.

### Preparation of Ac-MPS

The obtained dried mycelia powder by liquid fermentation technology^16^ was extracted with five-fold volumes of hydrochloric acid (1 mol/L) for 6 h at 40 °C (w/v). After centrifugation (3000 rpm, 10 min), the supernatant homogenate was precipitated with three volumes of ethanol (95%, v/v) overnight (4 °C). The precipitates were collected by centrifugation (3000 rpm, 10 min), and deproteinated by the method of Sevag^17^, respectively. Finally, the purified precipitates were collected by lyophilization for further experiments.

### Structural characterization of Ac-MPS

The monosaccharide compositions were analyzed by gas chromatography (GC, GC-2010, Shimadzu, Japan) equipped with a capillary column of Rtx-1 (30 m × 0.32 mm × 0.2 μm) using the reported method^18^. The samples and standard monosaccharides were hydrolyzed by trifluoroacetic acid (TFA) at 120 °C for 4 h, and the residual TFA was removed with methanol. The hydrolyzate was acetylated by hydroxylamine hydrochloride (12 mol/L, 10 mL) and sodium borohydride-ammonium hydroxide (2%, 0.3 mL). After centrifugation (4000 rpm, 10 min), the acetylated-hydrolyzate (1 μL) was injected into a capillary column of Rtx-1. The relative molar ratios of monosaccharide were investigated by the area normalization method according to the standard chromatograms of rhamnose, fucose, ribose, arabinose, xylose, mannose, galactose, and glucose.

The molecular weight of Ac-MPS is determined by high-performance gel permeation chromatography (HPGPC) using an Shodex SB-806HQ column (8 mm × 300 mm, Showa Denko K.K., Tokyo, Japan) in a HPLC system (Agilent 1260, Agilent Technologies, CA, USA). The calibration curve was constructed by a series of standard dextrans (Sigma) with different weight-average molecular weights (9.75, 36.8, 135.35, 300.6 and 2000 kDa), and the molecular weights were analyzed using Agilent GPC software.

The fourier transform infrared (FT-IR) spectra for Ac-MPS was determined with a Thermo-Nicolet 6700 spectrometer (Thermo Scientific, USA) in the range of 4000-400 cm^−1^.

The molecular morphology of Ac-MPS was observed by ScanAsyst of Atomic Force Microscope (AFM, BioScope Catylyst NanoScope V, Bruker, Billerica, MA). The samples (10 mg) were dissolved by 1 L distilled water, and then 5 μL of the diluted solution was dropped onto a freshly cleaved mica substrate and dry at room temperature. All images were acquired in tapping mode with 256 × 256 pixels at a scanning rate of 1.0 Hz per line.

### Ethics statement

All experiments were performed in accordance with the guidelines and regulations of the ethics committee of the Shandong Agricultural University and the Animals (Scientific Procedures) Act of 1986 (Amended 2013).

All experimental protocols were submitted to and approved by the ethics committee of the Shandong Agricultural University in accordance with the Animals (Scientific Procedures) Act of 1986 (Amended 2013).

### Acute toxicity study

The acute toxicity test was performed according to the method of Chao *et al*.^19^. Fifteen male Kunming strain mice (aging 8–10 weeks and weighing 18–22 g) were randomly divided into three groups (five in each group) gavaged with Ac-MPS at dosages of 600, 900 and 1200 mg/kg body weight (bw), respectively. The experiment was lasted for 40 consecutive days. All the mice were observed continuously for gross behavioral changes, toxic symptoms and mortality during the whole feeding period.

### Animal experiments

The HFE composed of oil phase including cholesterol (10 g), liquid lard oil (25 g), methylthiouracil (1 g) and of tween-80 (25 mL), as well as water phase including distilled water (30 mL), propylene glycol (20 mL) and sodium deoxycholate (2 g) was prepared according to the previous report^20^. The oil phase and water phase were freshly blended before animal administration.

The Kunming strain mice (male, 20 ± 2 g) with the lot No. of SCK(*Lu*)20140007 were purchased from Taibang Biological Products Ltd. Co. (Taian, China) and housed in cages under controlled conditions of 12 h light/dark cycles at 22 ± 2 °C and 60–65% humidity with free access to water and standard food. After adaptation for 7 days, all mice were randomly divided into six groups (ten in each group) including one normal control (NC) group, one model control (MC) group, one positive control (PC) group, as well as three dosage groups including one high dosage group (400 mg/kg bw), one middle dosage group (200 mg/kg bw) and one low dosage group (100 mg/kg bw). During the experiment procedure, the gavage of HFE and polysaccharides in dosage groups were processed alternately, using saline solution and simvastatin (200 mg/kg bw) as control in NC and PC groups, respectively. The experiment was lasted for 40 consecutive days, and at the end of the procedure, all mice were sacrificed by exsanguinations under diethyl ether anesthesia after fasting for 12 h.

Blood samples were collected in tubes and centrifuged at 14,000 rpm for 10 min using a refrigerated centrifuge at 4 °C to obtain the serum. The levels of total cholesterol (TC), triglyceride (TG), low-density lipoprotein cholesterol (LDL-C) and high-density lipoprotein cholesterol (HDL-C) in serum were measured using automatic biochemical analyzer (ACE, USA).

The liver, heart, kidney and spleen were rapidly isolated, weighed (the index was calculated by organic weight/bw ×100) and homogenized (1:9, w/v) immediately in phosphate buffer solutions (PBS, 0.2 mol/L, pH 7.4). After centrifugation (5,000 rpm, 4 °C) for 20 min, the supernatants were collected for further biochemical analysis. The activities of SOD, GSH-Px and CAT, as well as contents of MDA in liver, heart, kidney and spleen homogenates were determined by the commercial reagent kits according to the instructions.

Fresh tissues (liver, heart, kidney and spleen) were fixed in 4% formaldehyde solution overnight, embedded in paraffin, cutted in slices, and stained with hematoxylin and eosin. The slices were photographed under microscope showing the histopathological changes (×600 magnifications).

### Western bolt assay

The liver tissue lysates were prepared by homogenized in ice-cold lysis buffer (50 mmol/L HEPES, 10 mmol/L EDTA, 100 mmol/L NaF, 50 mmol/L Na pyrophosphate, 10 mmol/L Na Orthovanadate, and 1% Triton at pH 7.4), supplemented with protease/phosphatase inhibitor cocktails. The lysates were centrifuged 14,000 rpm for 10 min at 4 °C and the supernatant was collected. The protein concentration in the supernatant was determined by BCA. The primary antibodies p-AMPK (Cell Signaling Technology), and GAPDH (Abcam) were used at a dilution of 1:1000.

### Statistical analysis

The data were presented as the Means ± Standard Deviations (S.D.) from three independent experiments. Significant differences between the experimental groups were determined by the t-test and *P* < 0.05 was considered to be statistically significant.

## Results

### Structural characterization

**Figure 1:**
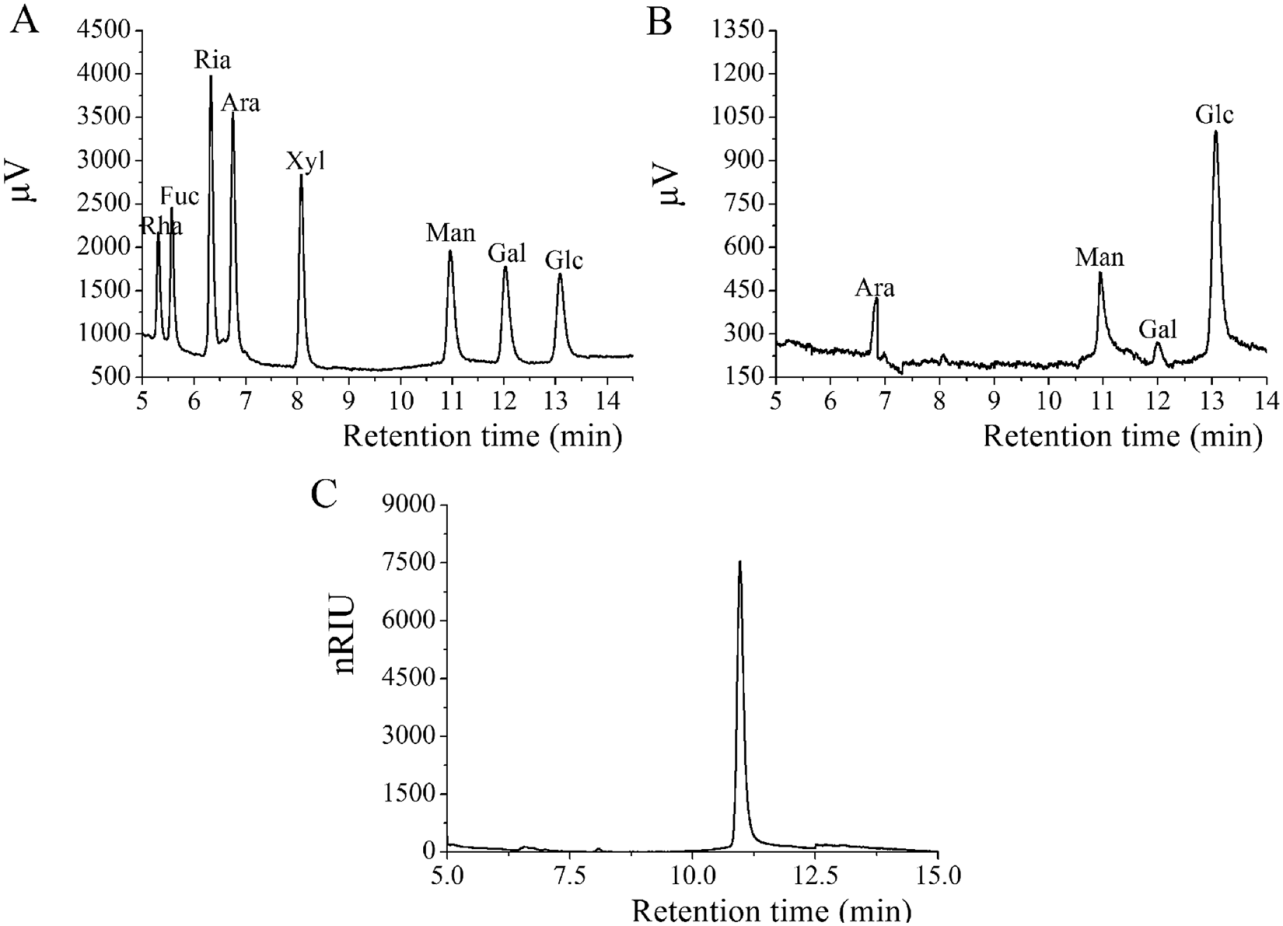
GC chromatograms of monosaccharides. (**A**) Standard sugars, and (**B**) Ac-MPS and HPGPC chromatograms of Ac-MPS (**C**).

Comparing with the retention time of the standards, the Ac-MPS was composed of arabinose, mannose galactose and glucose with a percentage composition of 10.7%, 23.2%, 13.4%, and 52.7% with the molar ratio of 7.99: 14.43: 8.33: 32.75 (Fig. 1). These data suggested that glucose was predominant monosaccharides in Ac-MPS. Furthermore, the molecular weight of Ac-MPS was analyzed by HPGPC and monitored with a differential refractive index detector (RID) as described. As showed in Fig. 1C, the Ac-MPS exhibited a narrow and symmetrical peak on HPGPC, indicating that Ac-MPS is homogeneous polysaccharide with an average molecular weights (Mw) of 2.712 × 10^5^ Da.

In the present work, the FT-IR was used to explore the formation of Ac-MPS, and the result exhibited the characteristic IR absorption of the polysaccharide at 1323 cm^−1^ and 1641 cm^−1^. Moreover, the broad stretching intense characteristic peak at around 3431 cm^−1^ was mainly due to the hydroxyl group^21^, and a weak C-H stretching band showed at 2928.54 cm^−1^. The peak at the range of 1100–1010 cm^−1^ (1079.75 cm^−1^ and 1051.06 cm^−1^) indicated the possible presence of furanose ring in polysaccharides^22^.

**Figure 2:**
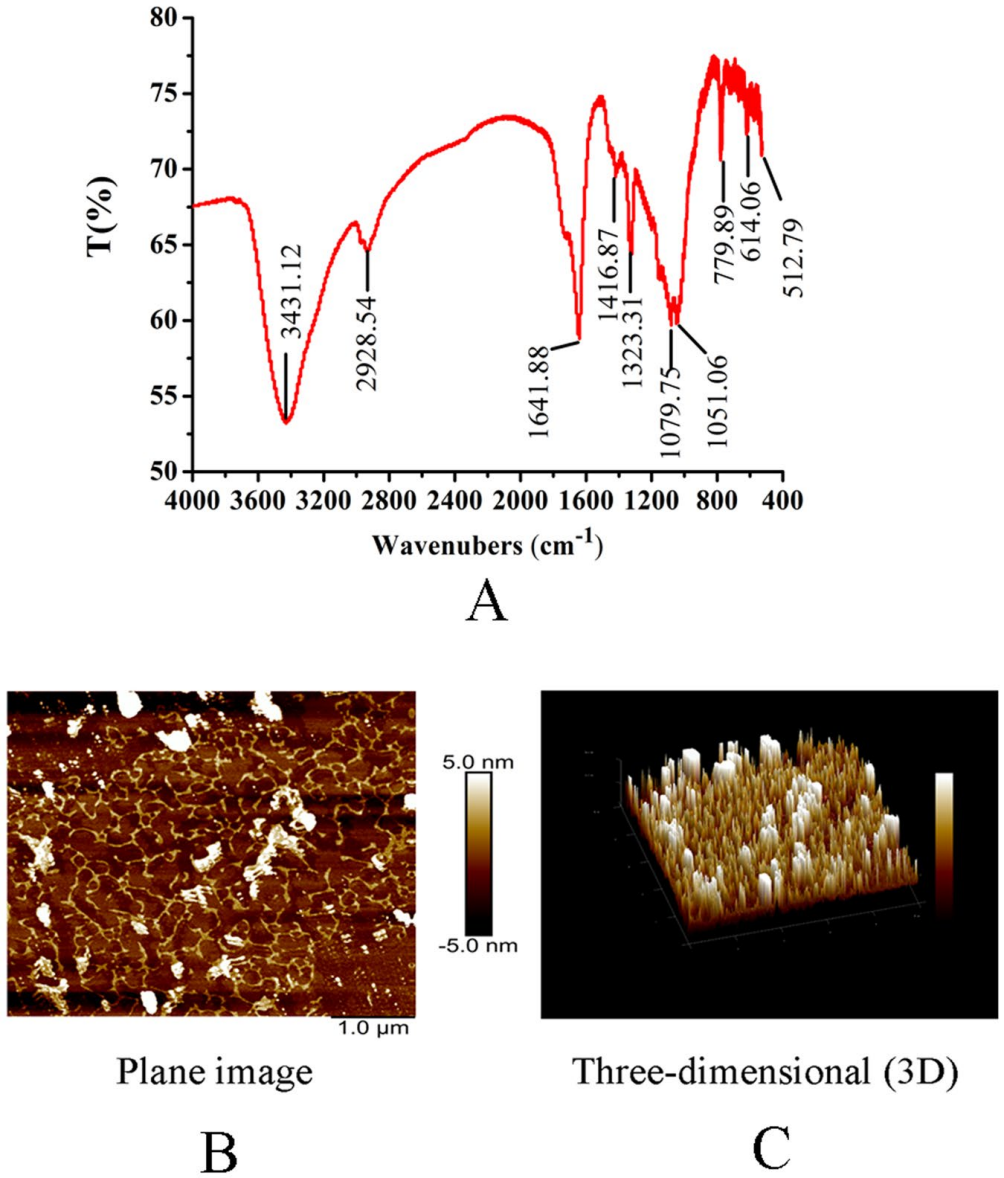
The FT-IR spectra (**A**) and AFM image of Ac-MPS (**B**,**C**).

The spatial structure and surface morphology of Ac-MPS was evaluated by AFM and the results including plane and three-dimensional (3D) were shown in Fig. 2. The planar morphology showed that Ac-MPS possess the multiple-branching construction with a few irregular aggregation. The 3D micrograph also showed the extended shapes and accumulative degree of Ac-MPS chains. The heights and widths of branches were during in the ranges of 0.5–4.5 nm and 18–35 nm, respectively.

#### Acute toxicity studies

The mice treated with Ac-MPS even at the ultimate dose of 1200 mg/kg bw did not appear significant changes in behavioral, autonomic and toxic responses. Meanwhile, no deaths were observed at the end of the experiment and during the investigation period. These results indicated that Ac-MPS was practically non-toxic substances.

#### Effects of Ac-MPS on bw and organic index

The effects of Ac-MPS on bw, as well as index of liver, heart, kidney and spleen in HFE-induced hypertriglyceridemic mice were presented in Table 1. Initially, the bws in all groups were non-differences. However, after 40 days of experiment, the bw of MC group (41.80 ± 0.67 g) showed significantly higher than that in NC group (33.70 ± 0.64 g). Satisfactorily, after the treatment of Ac-MPS at three dosages, the bws were declined by 16.5 ± 1.57%, 12.7 ± 2.12% and 6.7 ± 1.67% when compared with the mice in MC group, indicating that the Ac-MPS had potential and dose-dependent contributions in suppressing the weight gain induced by HFE.

**Table 1:** Effects of Ac-MPS on bws and organic index in HFE-induced hypertriglyceridemic mice

Groups	Bws (g)		Organic index (%)			
	Initial	Final	Liver	Heart	Kidney	Spleen
NC	30.30 ± 0.46	33.70 ± 0.64	4.82 ± 0.26	2.08 ± 0.06	3.15 ± 0.27	1.94 ± 0.17
MC	31.30 ± 0.50	41.80 ± 0.67^a^	7.56 ± 0.34^a^	3.45 ± 0.27^a^	5.11 ± 0.28^a^	3.04 ± 0.29^a^
PC	30.10 ± 0.47	35.04 ± 0.72^b^	4.98 ± 0.30^b^	2.25 ± 0.20^b^	3.21 ± 0.21^b^	1.99 ± 0.15^b^
400 mg/kg bw	31.10 ± 0.48	34.80 ± 0.66^b^	4.91 ± 0.31^b^	2.31 ± 0.27^b^	3.17 ± 0.15^b^	2.01 ± 0.11^b^
200 mg/kg bw	30.89 ± 0.39	38.80 ± 0.89^b^	5.28 ± 0.32^b^	2.53 ± 0.30^b^	3.82 ± 0.27^b^	2.63 ± 0.22^b^
100 mg/kg bw	30.70 ± 0.50	40.80 ± 0.70^b^	6.88 ± 0.43^ab^	3.00 ± 0.28^ab^	4.82 ± 0.32^b^	2.98 ± 0.27^b^

The values are reported as the Mean ± S.D. of five mice per group. ^a^*P* < 0.01 compared with NC group; ^b^*P* < 0.01 compared with the MC group.

Simultaneously, significant increases on organic index could be seen in MC group when compared with that in the NC group (*P* < 0.01, Table 1). However, the increases of index on liver, heart, kidney and spleen could be alleviated by intervention with Ac-MPS at three different doses (*P* < 0.01), respectively. Especially, in the high dosage group of Ac-MPS, the liver and heart index, which reached 4.91 ± 0.31% and 2.31 ± 0.27%, were almost approximate to that in the PC group.

#### Effects of Ac-MPS on serum lipids levels

**Figure 3:**
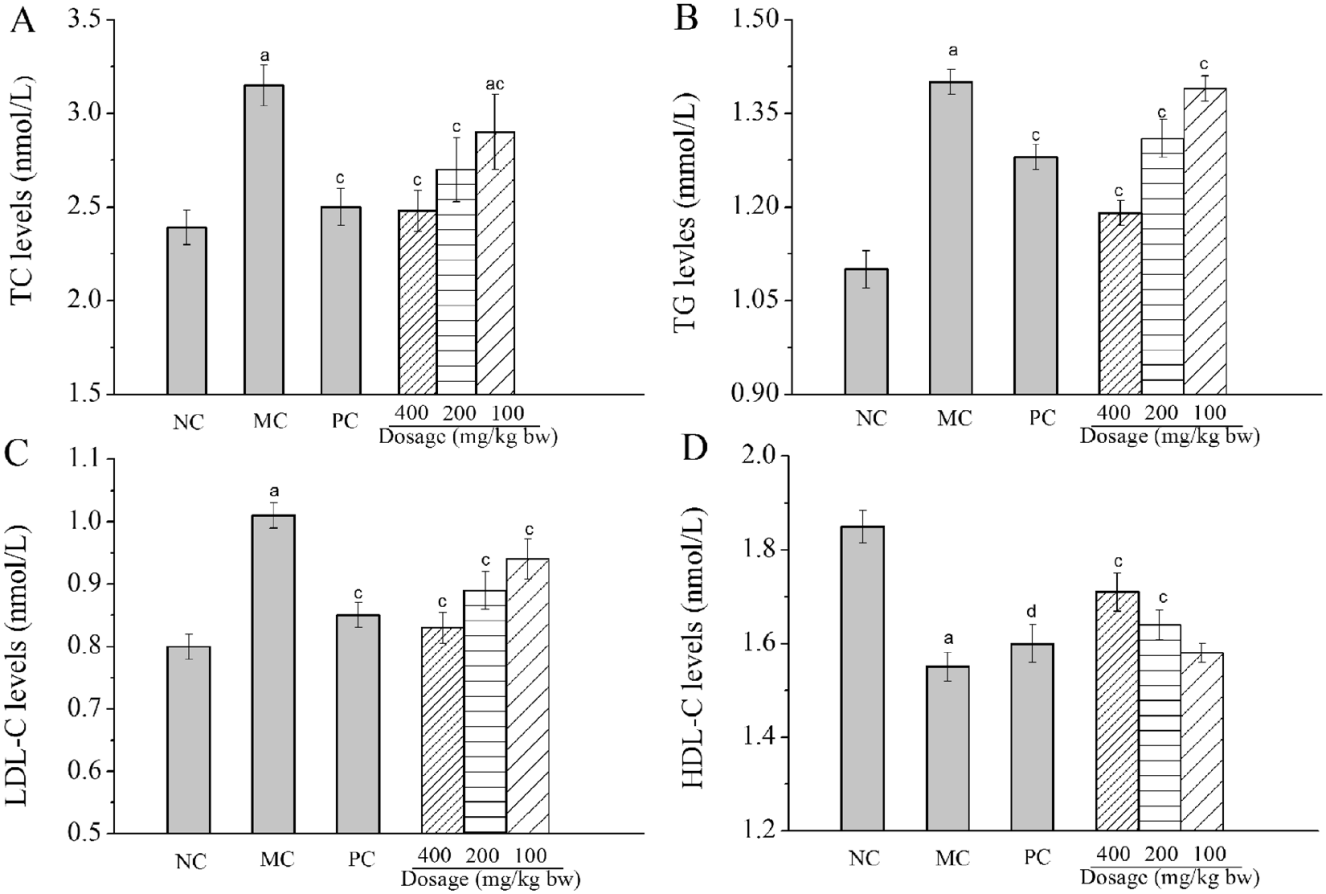
Effects of Ac-MPS on levels of TC (**A**), TG (**B**), LDL-C (**C**) and HDL-C (**D**) in HFE-induced hypertriglyceridemic mice. The values are reported as the Mean ± S.D. of five mice per group. (a) *P* < 0.01 compared with NC group; (c) *P* < 0.01 and (d) *P* < 0.05 compared with the MC group.

Clinically, the serum TC, TG, LDL-C and HDL-C levels were commonly used as biochemical markers on diagnosing the HTG. As shown in Fig. 3, when compared with that in the NC group, significant increases of TC, TG, and LDL-C levels, as well as decrease of HDL-C levels were observed in mice after the gavage with HFE (MC groups), revealing that the severe hypertriglyceridemia had been occurred. However, when compared with that in the MC group, the TC, TG and LDL-C levels reached 2.48 ± 0.11 mmol/L (Fig. 3A), 1.19 ± 0.02 mmol/L (Fig. 3B), and 0.83 ± 0.025 mmol/L (Fig. 3C) by the treatment with Ac-MPS at the dosage of 400 mg/kg bw, which were 21.27 ± 3.1%, 15.00 ± 1.4%, and 17.82 ± 2.4% lower than that in MC group (with all *P* < 0.01), respectively, while the HDL-C level reached 1.71 ± 0.041 mmol/L (Fig. 3D), which were 10.32 ± 2.6% higher than that in MC group at the same dosage. Besides, all dosage groups displayed dose-dependently. These results indicated that the Ac-MPS could significantly remit the HTG by recovering the levels of serum lipids.

#### Effects of Ac-MPS on SOD, GSH-Px, CAT, and T-AOC activities

As Table 2 displayed, significant decreases of SOD, GSH-Px, CAT and T-AOC activities were observed in HFE-induced HTG mice when compared with that in the NC group (*P* < 0.01), respectively. In the present work, the SOD, GSH-Px, CAT, and T-AOC activities of Ac-MPS in liver, kidney and spleen expressed dose-dependently. Nevertheless, the three dosages of Ac-MPS on these enzymes (SOD, GSH-Px, CAT, and T-AOC) showed no obvious dose effects in heart. As shown in Table 2, the SOD activities at 200 mg/kg bw treated with Ac-MPS reached 13.78 ± 0.49 U/mg prot (*P* < 0.01), with 0.95 ± 0.14% higher than that in the PC group. And the GSH-Px activities of mice in heart treated with Ac-MPS at the dosage of 400 mg/kg bw and 200 mg/kg bw were increased by 195.5 ± 5.37% and 174.0 ± 4.45% when compared with that in MC group, respectively. Besides, after the treatment with Ac-MPS at 400 mg/kg bw, the CAT and T-AOC activities reached 15.78 ± 0.48 U/mg prot and 0.132 ± 0.004 U/mg prot in heart, which were 43.7 ± 4.37% and 32.0 ± 4.0% higher than that in MC group.

**Table 2:** Effects of Ac-MPS on activities of SOD, GSH-Px, CAT and T-AOC in liver, heart, kidney and spleen.

	NC	MC	PC	Ac-MPS (mg/kg bw)		
				400	200	100
**Liver**						
SOD	11.40 ± 0.32	8.03 ± 0.26^a^	10.00 ± 0.21^b^	10.40 ± 03^b^	9.41 ± 0.25^b^	9.01 ± 0.24^b^
GSH-Px	2.13 ± 0.10	0.70 ± 0.06^a^	1.90 ± 0.08^b^	2.07 ± 0.09^b^	1.73 ± 0.08^a^	1.53 ± 0.08^b^
CAT	25.23 ± 0.78	12.07 ± 0.63^a^	20.00 ± 0.54^b^	19.76 ± 0.43^b^	17.44 ± 0.38^b^	14.81 ± 0.34^b^
T-AOC	0.24 ± 0.005	0.20 ± 0.006^a^	0.23 ± 0.004	0.22 ± 0.003^b^	0.22 ± 0.004^b^	0.21 ± 0.003^b^
**Heart**						
SOD	14.16 ± 0.45	9.03 ± 0.41^a^	13.65 ± 0.51^b^	11.17 ± 0.38^b^	13.78 ± 0.49^b^	9.97 ± 0.38^ab^
GSH-Px	18.35 ± 0.52	8.75 ± 0.65^a^	16.35 ± 0.45^b^	17.11 ± 0.47^b^	15.23 ± 0.39^a^	12.89 ± 0.33^b^
CAT	16.35 ± 0.55	10.98 ± 0.45^a^	15.35 ± 0.56^b^	15.78 ± 0.48^b^	13.21 ± 0.39^b^	11.78 ± 0.32^a^
T-AOC	0.15 ± 0.005	0.10 ± 0.006^a^	0.13 ± 0.005^b^	0.13 ± 0.004^b^	0.12 ± 0.005^a^	0.11 ± 0.003^b^
**Kidney**						
SOD	20.83 ± 1.11	10.61 ± 0.37^a^	18.88 ± 0.49^b^	19.01 ± 0.78^b^	17.27 ± 0.39^b^	13.33 ± 0.56^b^
GSH-Px	2.89 ± 0.17	0.81 ± 0.05^a^	2.11 ± 0.15^b^	2.21 ± 0.14^b^	1.88 ± 0.23^b^	1.19 ± 0.21^b^
CAT	28.73 ± 1.33	14.39 ± 0.89^a^	25.66 ± 1.67^b^	26.01 ± 2.01^b^	20.31 ± 1.86^b^	14.98 ± 0.91^a^
T-AOC	0.54 ± 0.04	0.15 ± 0.02^a^	0.43 ± 0.03^b^	0.45 ± 0.03^b^	0.37 ± 0.021^b^	0.21 ± 0.01^b^
**Spleen**						
SOD	13.69 ± 0.28	9.11 ± 0.22^a^	12.76 ± 0.31^b^	12.91 ± 0.3^b^	10.81 ± 0.22^b^	5.87 ± 0.27^b^
GSH-Px	22.49 ± 0.52	11.97 ± 0.48^a^	20.88 ± 0.49^b^	20.76 ± 0.48^b^	16.66 ± 0.39^b^	13.21 ± 0.38^a^
CAT	11.8 ± 0.58	7.17 ± 0.31^a^	10.76 ± 0.43^b^	9.8 ± 0.37^b^	8.31 ± 0.31^b^	7.33 ± 0.21^a^
T-AOC	1.00 ± 0.02	0.31 ± 0.008^a^	0.98 ± 0.03^b^	0.87 ± 0.021^b^	0.51 ± 0.011^b^	0.39 ± 0.01^b^

The values are reported as the Mean ± S.D. of five mice per group. ^a^*P* < 0.01 compared with NC group; ^b^*P* < 0.01 compared with the MC group.

#### Effects of Ac-MPS on LPO and MDA contents

**Figure 4:**
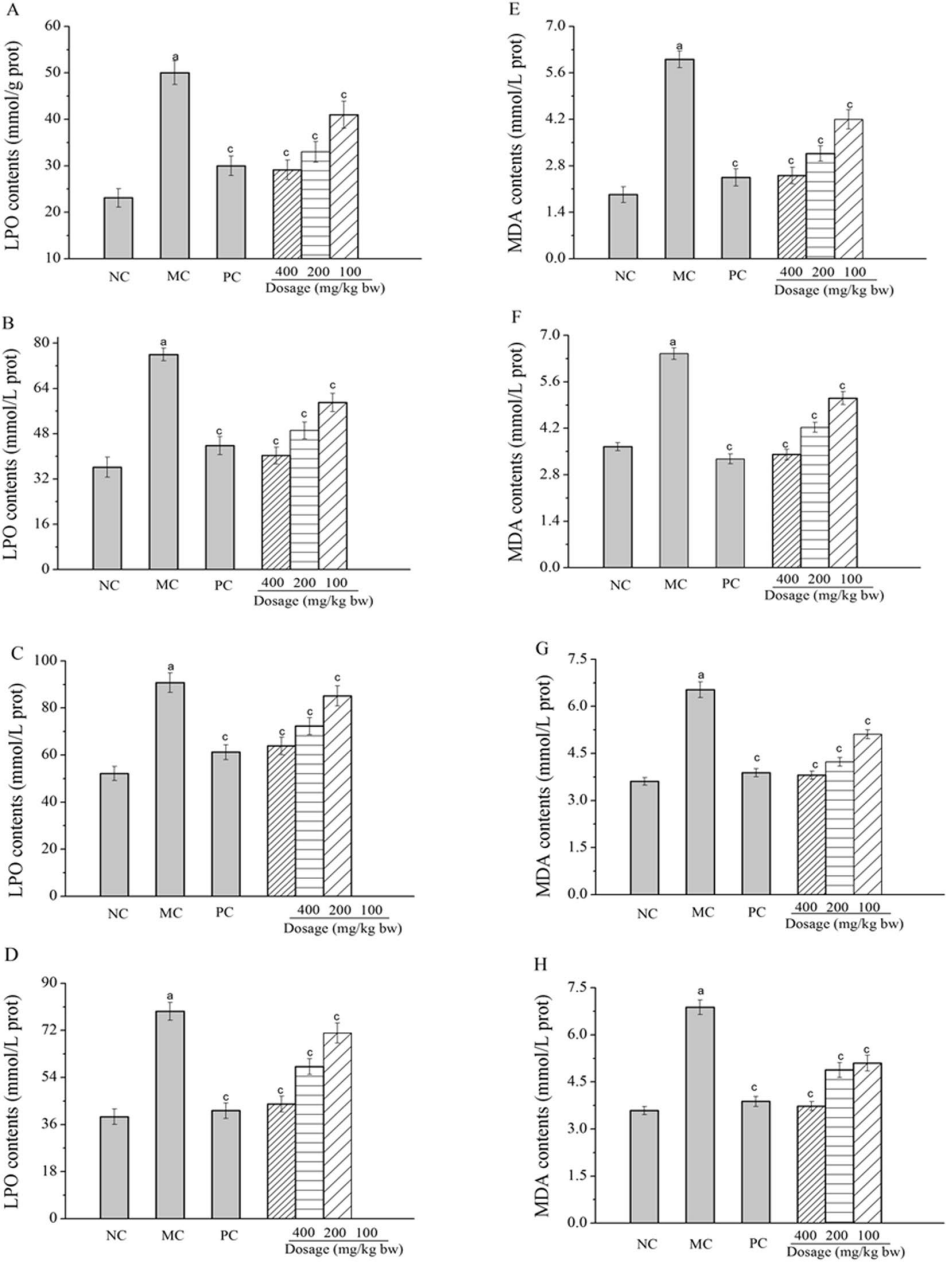
Effects of Ac -MPS on the contents of LPO, (**E**) MDA in liver (**A**), heart (**B**), kidney (**C**), and spleen (**D**), as well as MDA in liver (**E**), heart (**F**), kidney (**G**), and spleen (**H**) in HEF-induced hypertriglyceridemic mice. The values are reported as the Mean ± S.D. of ten mice per group: (a) *P* < 0.01 compared with NC groups; (c) *P* < 0.01 compared with the MC groups.

As illustrated in Fig. 4, after the treatment with HFE, significant enhancement of LPO and MDA contents could be seen in organic homogenate in MC groups when compared with that in NC group (*P* < 0.01). Interestingly, the LPO contents of mice treated with Ac-MPS at 400 mg/kg bw reached 29.13 ± 2.11, 40.27 ± 2.97, 63.87 ± 3.77 and 43.81 ± 3.03 mmol/g prot in liver, heart, kidney and spleen, with about 41.7 ± 4.21%, 47.0 ± 3.91%, 29.6 ± 4.15% and 44.7 ± 3.82% lower than that of MC group (50.03 ± 2.56, 76.01 ± 2.23, 90.75 ± 4.17 and 79.31 ± 3.45 mmol/g prot). Similarly, the MDA contents reached 2.51 ± 0.25, 3.41 ± 0.16, 3.81 ± 0.13 and 3.73 ± 0.14 mmol/g prot in liver, heart, kidney and spleen of mice treated with Ac-MPS at the dosage of 400 mg/kg bw, respectively (Fig. 4E–H). Thus, we could deduce that Ac-MPS had strong potential antioxidant effect at high dose (400 mg/kg bw) on liver, heart, kidney and spleen.

#### Signaling pathways involved in AMPK induction by Ac-MPS

**Figure 5:**
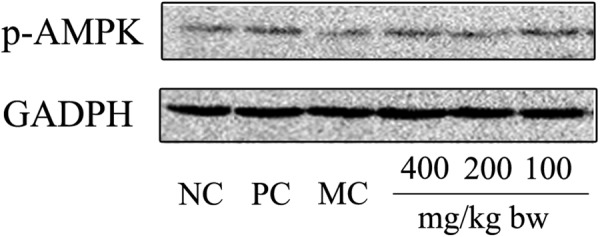
Western blot analysis of the levels of p-AMPK proteins.

We take further investigation to elucidate how Ac-MPS induced the production of phosphorylated AMPK protein levels in HTG-mice. As shown in Fig. 5, the p-AMPK protein levels were significantly lower in the MC group than that in the NC group. However, mice in the Ac-MPS intervened group, especially at the high dose group, showed an increase in the phosphorylation levels of AMPK proteins compared to those observed in the MC group.

#### Histopathological analysis

**Figure 6:**
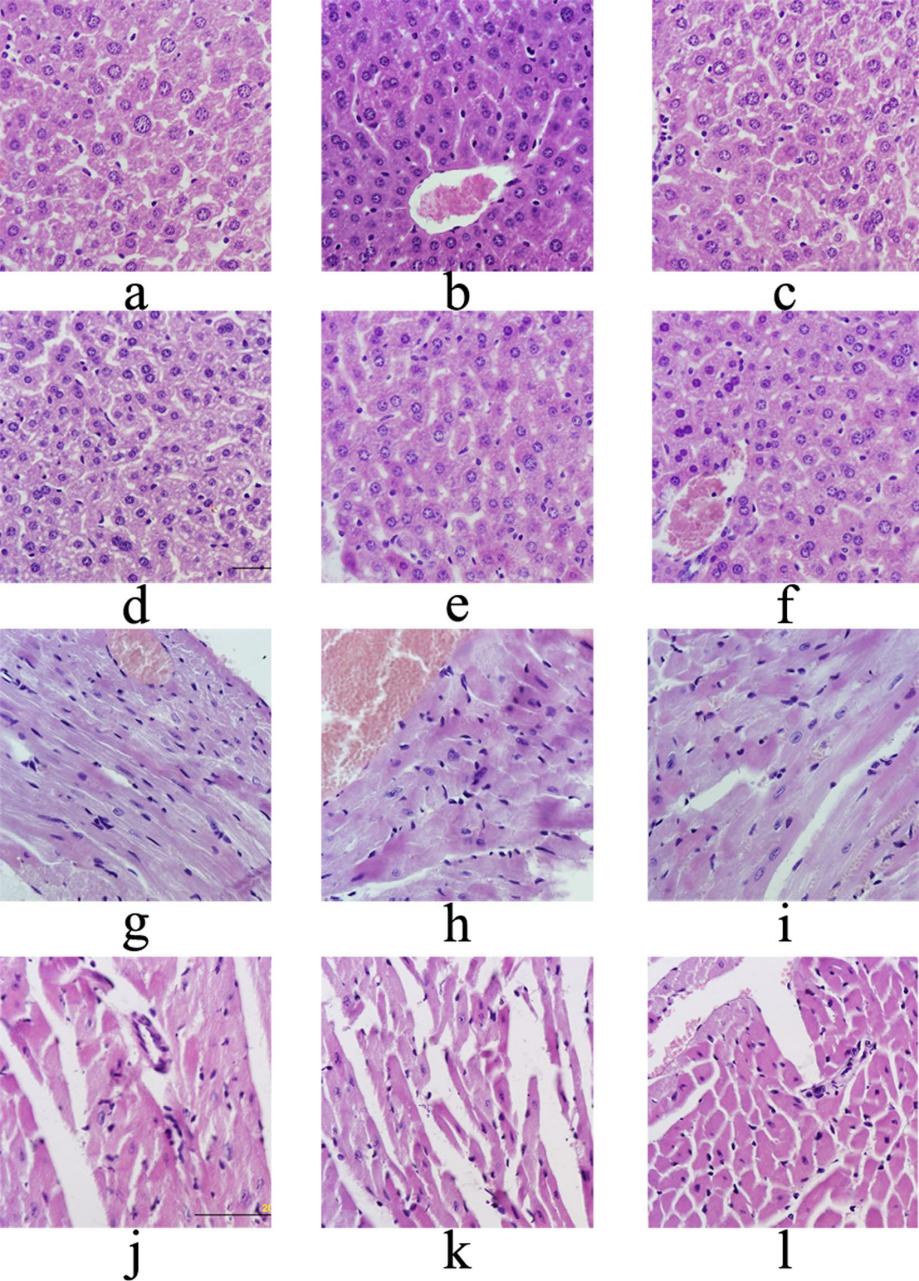
Optical micrographs of mice liver and heart tissues slice (magnification 600×). (**a**) Liver of mice in NC groups, (**b**) liver of mice in MC groups, (**c**) liver of mice in PC groups, (**d–f**) liver of mice fed with Ac-MPS at three doses; (**g**) heart of mice in NC groups, (**h**) heart of mice in MC groups, (**i**) heart of mice in PC groups, (**j–l**) heart of mice fed with Ac-MPS at three doses.

**Figure 7:**
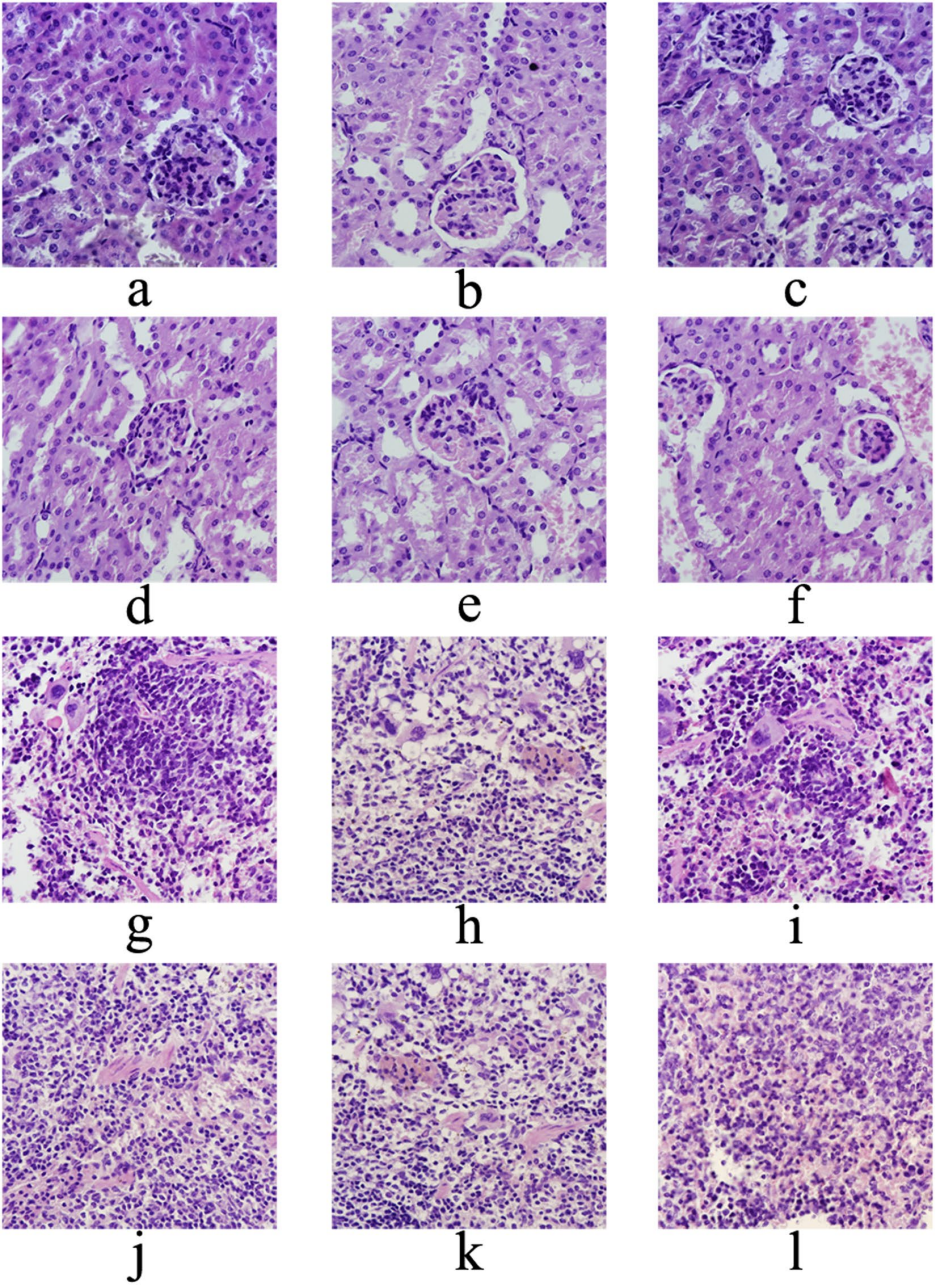
Optical micrographs of mice kidney and spleen tissues slice (magnification 600×). (**a**) Kidney of mice in NC groups, (**b**) kidney of mice in MC groups, (**c**) kidney of mice in PC groups, (**d–f**) kidney of mice fed with Ac-MPS at three doses; (**g**) spleen of mice in NC groups, (**h**) spleen of mice in MC groups, (**i**) spleen of mice in PC groups, (**j–l**) spleen of mice fed with Ac-MPS at three doses.

The effects of Ac-MPS on liver, heart, kidney and spleen histopathology of HEF-treated mice were presented in Figs 6 and 7. Obviously, compared with the normal cells in regular morphology with an abundant cytoplasm, distinct nuclei, and orderly arranged cell cords (Figs 6 and 7), the tissues of mice in the MC group showed some degree of damage and pathological changes especially in liver and kidney. As displayed in Fig. 6b, the extreme cellular swelling, large fat vacuoles accumulation, and the disappearance of nuclei were found in MC group, indicating that the obvious hepatic steatosis, necrosis, and vesicular degeneration were occurred in the liver. In the way that we expected, those pathological changes were markedly ameliorated by the pretreatment with Ac-MPS at different dosages (100, 200 and 400 mg/kg bw). Meanwhile, as showed in Fig. 7b, the kidney in MC group showed severe kidney damage of destruction at glomerulus, inflammatory cell infiltration, lesions of tubulointerstitial, swelling of the kidney tubular epithelial cells, and the expansion glomerular interstitial, while treated with Ac-MPS, the trend of deterioration were retarded. And after treated by the Ac-MPS, the pathological symptoms with a certain degree of improvement were also observed in heart and spleen.

## Discussions

The polysaccharides, as the natural substance from medicinal mushrooms, had demonstrated to show effects on remitting the HTG significantly^23^. However, for the mycelium cell wall are dense and hard-degradation, the traditional extraction method with many insufficient problems of long extraction time, high extraction temperature, high energy consumption and low extraction yield is need to be improved^15^. And the acidic-assisted extraction, had been received more and more academic attentions due to its high extraction yield, reproducibility and simplified manipulation^24^. Meanwhile, previous literatures had indicated that the acidic-polysaccharides showed superior effects on possessing antioxidant activities^25^. Additionally, the hypertriglyceridemia had been incriminated as a fatal factor in inducing fatty liver, hypertension, atherosclerosis and cerebrovascular disease^2–4^. Besides, Dong-Won Shin *et al*. demonstrated that the hypertriglyceridemia is associated with diabetes and Kevin R *et al*. had pointed out that severe hypertriglyceridemia could lead to splenomegaly^26,27^. Hence, in present work, the protective effects of Ac-MPS from *P. eryngii* var*. tuoliensis* against HTG and its complications on liver, heart, kidney and spleen were investigated.

The high-fat emulsion (HFE), acted as chemical agents that caused the disordered circulatory of lipoproteins, was the immediate reason for increasing the TC, TG and LDL-C levels and decreasing the HDL-C levels. And it is well characterized to establish a hypertriglyceridemia model for evaluating the therapeutic potential effects of lipid metabolic disorder disease^20,28^. Moreover, as an ideal chemical drug for modeling hypertriglyceridemia, it is widely used in several animals, including mice^29^. Clinically speaking, the abnormal increase of LDL-C, TC, and TG levels, as well as the decrease of serum HDL-C levels, were fatal in increasing the blood viscosity, which was the premonition of atherosclerosis and cardiovascular diseases^30^. It had been demonstrated that the excess LDL-C, which was the main carrier of TC and TG to peripheral tissues, could be aggregated at the blood vessel walls, easier in causing the formation of atherosclerotic plaque lesion and coronary heart diseases^31^. However, as an advantageous lipoprotein, physiological-high HDL-C levels could transport the TC from the peripheral tissues to the liver for catabolism by the “reverse cholesterol transport” pathway during the blood circulation, reducing the lipid levels in serum^32^. In addition, previous reports had indicated that increased bw was associated with the severe HTG^32^. In the present work, the levels of TC, TG, LDL-C and the bw were increased obviously, and the levels of HDL-C were decreased significantly in the hypertriglyceridemia mice induced by the gavage of HFE, which indicated the severe hypertriglyceridemia was successfully established in mice.

Furthermore, in order to analyze the oxidative stress on HTG-induced organic damages and investigate the protective effects of the Ac-MPS on liver, heart, kidney and spleen, the activities of antioxidant enzymes (SOD, GSH-Px, CAT and T-AOC) and the lipid contents (MDA and LPO) were determined. Previous literatures had pointed out that the hypertriglyceridemia was accompanied with the destruction of antioxidant enzyme defenses *in vivo*, which could lead the cell and tissue damages^33^. Antioxidant enzyme defenses including SOD, GSH-Px and CAT were the first line of defense against oxidative injury in mammalian systems^34^. The possible mechanism might be that SOD could catalyze superoxide radicals to form hydrogen peroxide, and GSH-Px as well as CAT could catalyze hydrogen peroxide to water, hence the formation of ROS was prevented^35^. Moreover, the non-enzymatic antioxidant capacity against various reactive oxygen radicals and the increased susceptibility to oxidative damage could reflect by T-AOC activities^31^. In addition, the lipid peroxidation (LPO and MDA) could be deemed to the hallmark of oxidative stress causing tissue damages and it reflected the contents of free radicals produced by lipid peroxidation^36^. In our study, severe decreases in SOD, GSH-Px, and CAT activities, as well as increases in MDA and LPO contents were induced in hypertriglyceridemic mice when compared with that in the NC group, suggesting that oxidative damage had been occurred in the liver, heart, kidney and spleen. After pretreatment with Ac-MPS at three tested dosages, these pathological alterations were remitted significantly, with the potential mechanism of the scavenging capabilities of Ac-MPS on ROS. Similar results were expressed by Vamanu about *Pleurotus ostreatus* polysaccharides^37^.

To assess the regulatory mechanism of Ac-MPS in molecular biology, western blot for AMP-activated protein kinase (AMPK) was applied. AMPK is an energy sensor involved in lipogenesis, fatty acid oxidation and plays vital roles in regulating adipocyte metabolism^38^. AMPK phosphorylates and activates the Acetyl-CoA carboxylase (ACC), resulting in decreased production of malonyl-CoA, stimulating the β-oxidation of the fatty acids^39^. Additionally, the activation of the AMPK pathway inhibits 3-hydroxy 3-methylglutaryl coenzyme A reductase (HMGCR), which is the rate-limiting enzymes for cholesterol biosynthesis^40^. In our work, the p-AMPK protein levels had been markedly improved by Ac-MPS at 400 mg/kg bw, indicating that the Ac-MPS could alleviate hypertriglyceridemia by activating the AMPK via phosphorylation. These results are in concordance with previous reports that polysaccharides exert lipid metabolism regulation partly by inducing fatty acid oxidation and transport and reduce lipid biosynthesis to ameliorate hypertriglyceridemia by enhancing AMPK activation^39^.

In order to express the injury of tissues visually, hematoxylin-eosin staining was applied in this study. And the teratocyte, as the common features of organ lesions^41^, could be seen in the histopathological observation of MC group. However, these histopathological changes were significantly attenuated by Ac-MPS at three dosages, especially the treatment with Ac-MPS at 400 mg/kg. Hence, Ac-MPS might be useful as an adjuvant therapy for inhibiting the progression of hypertriglyceridemia.

As we known that the antioxidant properties of polysaccharides were mainly associated with their characterizations^42^. In the present work, the major monosaccharide composition of Ac-MPS with the multiple-branching construction and a few irregular aggregation was glucose and the FT-IR spectroscopy revealed that the furanose ring was presented in Ac-MPS. Compared with other literature, the similar result was expressed in *P. ostreatus* polysaccharides and *P. eryngii* polysaccharides^37,43^. But the antioxidant activity of Ac-MPS was higher than that isolated from *P. eryngii* and *P. ostreatus* polysaccharides. The differences of antioxidant activity possibly resulted from different species of *Pleurotus*, the acid-assisted extraction technologies and the analysis methods for the polysaccharides.

## Conclusions

The Ac-MPS with major component of glucose and presented furanose ring had potential antioxidant activities and impressive protective effects against HTG-induced organic damages, suggesting that the Ac-MPS could be used as potentially natural and functional foods for the prevention and alleviation of severe HTG and its complications.

## Electronic supplementary material

Supplementary Information
