# Supplementary material for: The characteristic, antioxidative and multiple organ protective of acidic-extractable mycelium polysaccharides by Pleurotus eryngii var. tuoliensis on high-fat emulsion induced-hypertriglyceridemic mice
Source: Sci Rep. 2018 Nov 30;8:17500. doi: 10.1038/s41598-018-35706-8 (PMC6269502; doi:10.1038/s41598-018-35706-8)
Supplement: Supplementary file 1 — Supplementary Information [file 41598_2018_35706_MOESM1_ESM.doc]

**The characteristic, antioxidative and multiple organ protective of acidic-extractable mycelium polysaccharides by *Pleurotus eryngii* var. *tuoliensis* on high-fat emulsion induced-hypertriglyceridemic mice**

*Zheng Gao ^1^, Qiangqiang Lai ^1^, Qihang Yang ^1^, Nuo Xu ^1^, Wenbo Liu ^1^, Fulan Zhao ^2^, Xinchao Liu ^1^, Chen Zhang ^1^, Jianjun Zhang 1, [[1]](#footnote-2)*, Le Jia 1, [[2]](#footnote-3)**

*^1^ College of Life Science,* *Shandong Agricultural University, Taian, 271018, PR China*

*2 The First People's Hospital of Taian, Taian, 271000, PR China*

**Supplementary information total gel**

**Figure S1**

The p-AMPK blot cropped from below gel and the GAPDH used as control.


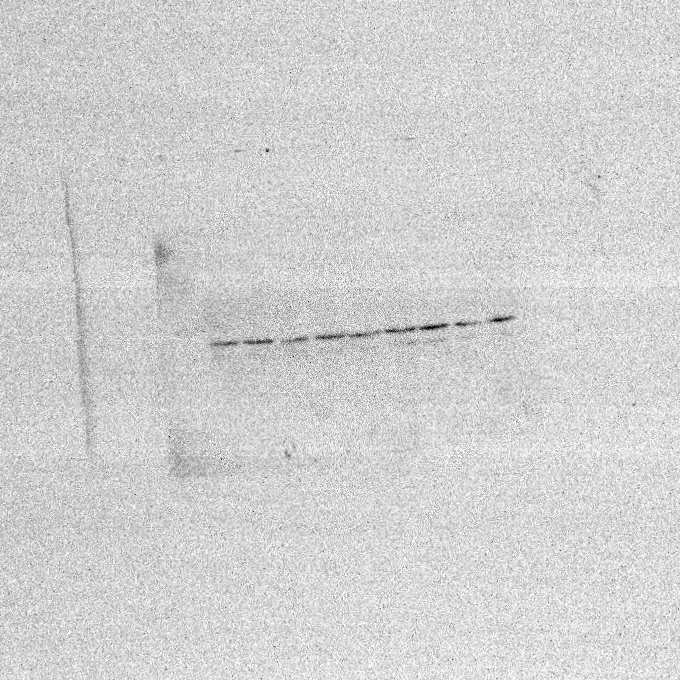


**The raw western bolt of p-AMPK**

(The first six imprints were used in the experimental results, and the remaining three holes were used as duplications of the experimental group)


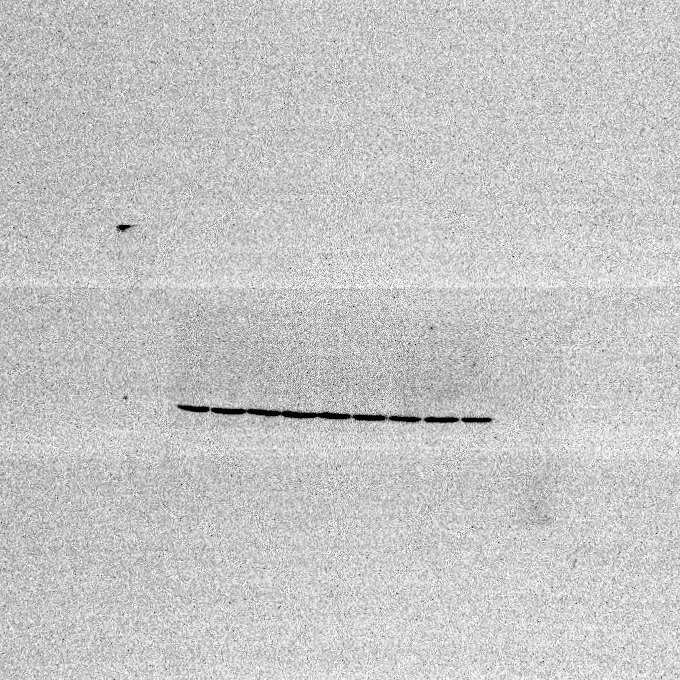


**The raw western bolt of GAPDH**

(The first six imprints were used in the experimental, and the remaining three holes were used as duplications of the experimental group)

1. ^*^E-mail address: jiale_446@163.com (L. Jia); yyxf0315@163.com (J.J. Zhang) [↑](#footnote-ref-2)
2. ^*^ [↑](#footnote-ref-3)
